# Quitting one’s job or leaving one’s profession: unexplored consequences of workplace violence and discrimination against health professionals

**DOI:** 10.1186/s12913-023-10208-0

**Published:** 2023-11-14

**Authors:** Oliver Hämmig

**Affiliations:** Epidemiology, Biostatistics and Prevention Institute of the University of Zurich, Hirschengraben 84, 8001 Zurich, Switzerland

## Abstract

**Background:**

Although workplace violence and discrimination against healthcare workers are global and universal phenomena, and violence at work is recognized as a serious and growing problem, in Switzerland, hardly anything is known about the related consequences on job changes and career endings, which are two major staffing challenges present in the notoriously understaffed healthcare sector.

**Method:**

Data collected from a written survey conducted among 1,840 hospital employees, of which 1,441 were health professionals, were used to evaluate and estimate the prevalence and impact of specific and cumulated forms of workplace violence and discrimination on the work climate and particularly on subsequent turnover intentions and career endings. Established multi- and single-item measures were used as predicting, intervening and outcome variables. Relative frequencies stratified for nurses, physicians and therapists were calculated to estimate and differentiate the size of the phenomena under study. Furthermore, crosstabulations, as well as multivariate regression analyses, were performed to explore the associations of interest.

**Results:**

Every fifth to sixth nurse and every seventh to eighth physician reported having had intentions to change jobs or leave the profession within the past year. These intentions become much more prevalent across all health professions when one or even two or more different forms of violence and/or discrimination at work are experienced and reported. Accordingly, the relative risks for intending to quit one’s job or leave one’s profession increase significantly and steadily with a growing number (1, 2 +) of different experienced forms of violence and/or discrimination at work compared to the reference group of those who are nonaffected (aOR from 2.5 up to 5.4). This fairly strong association was only slightly reduced (aOR from 2.1 to 4.0) when work climate was additionally taken into account as a potential intervening variable. Although work climate only partly accounted for the association under study, a poor work climate was an additional strong predictor and independent risk factor for intentions to turnover (aOR = 6.4) or leave the profession (aOR = 4.2).

**Conclusions:**

Experiences of workplace violence and discrimination and the resulting poor work climate both together and independent of each other seem to be important causes of job changes and career endings among healthcare workers in Switzerland.

## Background

### Job changes and career endings in healthcare

The shortage of nurses, physicians and other healthcare workers caused by those who are considering leaving direct patient care and medical/clinical practice and/or who are effectively doing so can be increasingly observed worldwide and recognized as a serious and growing problem [1–8]. Losing health professionals from the workforce in significant numbers is a major concern and challenge in a sector and setting that are notoriously struggling with problems of high drop-out and turnover, early retirement, premature career endings, staff shortages and chronic understaffing. Leaving the job and/or the profession or at least intending to do so is particularly and most frequently observed among nurses and physicians, i.e., in a typical female profession and an increasingly feminized profession, respectively, as reported by various studies from many countries. This has also been observed and reported in the case of Switzerland. An earlier cross-sectional study and survey conducted among hospital nurses and across various European countries, including Switzerland, namely, the RN4CAST study, revealed for Switzerland a proportion of 28% of nurses who intended to leave their current job and a proportion of 6% who intended to leave the profession within the next year [9]. A recent cohort study based on nationally representative register data has shown for Switzerland that approximately one in seven physicians has left patient care and medical/clinical practice since their graduation and that the probability of leaving patient care is increased considerably in physicians who graduated more recently [8].

In particular, hospitals are confronted with severe staff shortages and high fluctuations and turnover rates [10]. However, general practice is also facing a workforce “crisis” [5]. Health professionals in many cases engage in a stepwise withdrawal or exit by first reducing their hours or leaving the work unit, then quitting their job and changing their organization or health institution (hospital, nursing home, etc.); finally, they leave the (nursing, medical or other health) profession or clinical practice [2, 10]. As behaviour occasionally follows intention, it can reasonably be expected that actually leaving one’s job or profession is preceded by intending to do so. In other words, turnover intentions not always but sometimes will be put into practice.

The most studied and reported reasons for the decision or intention of health professionals to leave their organization or profession lie primarily and generally in poor and stressful working conditions; in high levels of demands and efforts and/or low resources and rewards at work and, as a result, in work overload, work-family conflict, reward frustration at work, job dissatisfaction and, above all, work stress; and related health problems such as burnout [2, 4, 6, 9, 11–17]. In contrast, social conflicts, aggression or unfair treatment at work among or against healthcare workers are issues that are much less recognized and studied as work stressors and potentially important reasons for intentions or decisions to leave a hospital or healthcare institution or even clinical practice and patient care as a career [18].

### Phenomena and consequences of violence and discrimination at work

Workplace violence and discrimination, similar to turnover intentions and behaviours and premature career endings, are global, serious and partly growing problems (especially in health professions) [18] that have received an increased amount of attention in international research independent of one another. Aggression and unfairness at work are not rare events or occasional occurrences but rather common and universal phenomena that are basically observed worldwide and in all economic sectors, i.e., across different countries, industries and occupations. However, workplace violence and discrimination are particularly observed in the healthcare sector, as reported by healthcare workers and most frequently observed and studied among hospital nurses (violence) and physicians (discrimination) [19–22]. Large-scale cross-sectional and systematic review studies from different countries have shown that sexual harassment, bullying and (non)physical violence among or against nurses and mistreatment, sexism and (gender) discrimination against physicians by patients or their families/visitors are quite prevalent phenomena [21, 22]; this is also true in Switzerland [23].

International research on this topic within the healthcare setting originates mainly from North American and Asian countries [19, 24] and focuses mainly on some health-related consequences. Consequently, the blind spots on the world map, in relation to the spectrum of occupations and the list of possible consequences, are numerous and large, and the dark figure is considered to be high. There are many unrecorded and unreported cases and unknown consequences of workplace violence or discrimination.

In European countries, particularly in German-speaking countries, little is known about the prevalence of workplace violence and discrimination against healthcare workers; furthermore, the related health- and work-related consequences have been poorly studied in health (services) research. In Switzerland, as in many other European countries, the problem is underreported and underestimated and therefore largely neglected or ignored and unexplored, except by very few studies [23, 25–28].

Losing empathy and distancing emotionally from patients (as symptoms of the burnout syndrome), being regularly absent from work for motivational and not health reasons (as the definition and indication of absenteeism) or intending to leave one’s job or one’s profession (as predictors of job changes, career endings and early retirements)—just to mention a few—are known and partly well-studied reactions and responses of healthcare workers to situations of chronic and high stress and strain at work (see i.a., [12, 14]). However, such exits, coping strategies and withdrawal behaviours are poorly explored as possible consequences of workplace violence and/or discrimination, with a few exceptions [1, 3, 29–35].

### Definitions of workplace violence and discrimination

Workplace violence is a collective term that includes basically any aggressive, abusive, harmful and/or violent behaviour experienced at work and stemming from clients, patients, relatives, visitors, work colleagues, supervisors, etc. Such violence is defined as “any act or threat of physical violence (including sexual assault), harassment, intimidation, or other threatening disruptive behaviour that occurs at the worksite with the intention of abusing or injuring the target” [36]. Workplace discrimination, on the other hand, describes any preference, disadvantage, unfairness or injustice experienced (or witnessed) at work and made, caused or committed by work colleagues, supervisors or employers on the basis of ascriptive, exclusive and not achievable attributes and on a personal or structural level. It is defined more specifically as the “unfair treatment and preferential (dis-)advantage of individuals or groups at work” [37] or as workplace-related injustice against workers [38] based on gender, age, disability, nationality, religion, race/colour, or sexual orientation. Workplace discrimination can occur at both an organizational or institutional level and at an individual or interpersonal level [37, 38].

These definitions clearly show that workplace violence and discrimination against (healthcare) workers are completely different phenomena that in turn include many different and very diverse incidents and behaviours, such as verbal abuse, physical aggression, sexual harassment, humiliation, bullying, mobbing and disadvantages due to age, sex, social status, body weight or shape, disability, nationality, ethnicity or race (skin colour), which stem from patients, relatives, colleagues and supervisors. In other words, they are more a hotchpotch or conglomeration of social problems and conflicts at work, with different parties involved, thus reflecting a collection of phenomena rather than a single problem and phenomenon. For this reason, individual aspects or forms of aggression and unfair treatment at work are usually studied in international research, mostly within individual occupations or health professions and not across different occupations. Workplace violence and discrimination are not studied in combination and as a common whole simply because such a concept does not exist, neither as a scientific construct nor as an isolated phenomenon.

### Research questions and hypotheses

As a consequence, and in contrast to previous studies from outside Switzerland, this study did not aim to examine individual aspects or forms of workplace violence or discrimination in individual and specific health professions (e.g., nurses, surgeons) or in particularly affected health institutions (e.g., psychiatric hospitals, nursing homes). Instead, the current study’s aim was to assess and investigate the general experience and existence (or presence) and the accumulation of any forms of violence and/or discrimination at work and the related effects on (or association with) job withdrawal intentions such as thoughts of changing one’s job or leaving one’s profession. Such intentions or thoughts of quitting or leaving have rarely and particularly in Switzerland never before been studied as possible consequences of stressful workplace violence and/or discrimination. Definitely there is not a single study in Switzerland as elsewhere in healthcare on effects of different accumulated and combined forms of aggression and unfair treatment experienced at work and from different sources. In addition, the current study aimed to examine these withdrawal intentions across different health professions and not just among nurses (violence) or among physicians (discrimination), as usually seen in the research literature.

Finally, the work climate was additionally considered in the study as a potential mediating or intervening variable since a good work culture or team climate has been found to be an important job resource and an effective preventive and protective factor against workplace violence and/or discrimination [3, 39–41]. It can be assumed therefore that workplace violence and/or discrimination only indirectly produce job changes or career endings since they initially worsen the work climate, and a poor work climate subsequently causes intentions (and actions) to quit one’s job and/or leave one’s profession. The question is whether and to what extent the work climate accounts for the relationship between experiences of workplace violence and discrimination and intentions to quit one’s job and/or leave one’s profession.

In sum, the present study tried to respond to the following three research questions on the basis of survey data collected among a reasonably large number of hospital employees and particularly health professionals in Switzerland:

1. How frequently do experiences of workplace violence and discrimination between and across different healthcare occupations occur? Are nurses more likely to experience (or report) violence, and are physicians more likely to experience (or report) discrimination?
2. Does an accumulation and combination of different experienced forms of violence and discrimination at work possibly increase the risk or likelihood of intentions to quit one’s job or leave one’s profession among health professionals?
3. Is this assumed association between experiences of violence and discrimination at work and intentions to quit one’s job or leave one’s profession partly or even largely mediated by a poor work climate, which is suspected to be the immediate effect of such experiences and the true cause for these intentions?

**Fig. 1:**
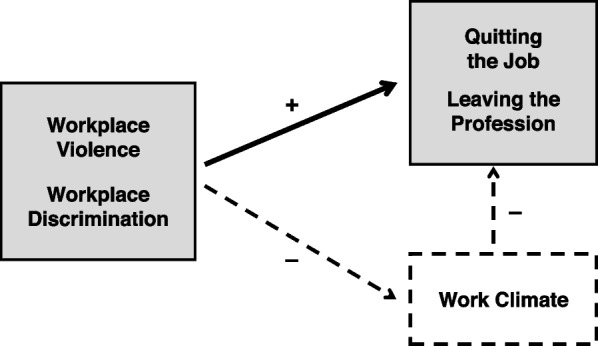
Theoretical model illustrating the hypothesized causal paths and analyzed associations and relations between the studied predictors or exposures (experiences of violence and discrimination at work), the outcomes of interest (poor work climate, turnover intention, intended career ending) and their interrelation

The current study’s research interest and analytical approach follow the assumed relationships between all involved factors or variables, as illustrated in Fig. 1.

## Methods

### Data and study sample

The present study is based on cross-sectional survey data collected in 2015 and 2016 among the workforces of a total of six public hospitals and rehabilitation clinics in German-speaking Switzerland and by means of a written and fully standardized questionnaire. The hospitals and clinics that were approached for participation in the postal survey were selected according to their type of institution (preferably a mix of cantonal, district and university hospitals and private hospitals/clinics) and the size of their workforce (at least 500 employees) and the language region where they are located (German-speaking part of Switzerland), with the aim of obtaining different kinds of hospitals and clinics and a sufficient sample size and number of cases to ensure enough statistical power for multivariate and/or stratified analyses. Due to operational and organizational reasons, a lack of time and the fear of obtaining survey results that would require consequences to be drawn, only approximately one in ten of the requested hospitals and clinics committed to participate. Although the hospitals and clinics were conveniently selected (first step), their employees were asked for survey participation (second step) on a completely anonymous and voluntary basis.

The full sample survey included one university hospital, one cantonal hospital, two district hospitals, and two rehabilitation clinics, with an aggregated total workforce of approximately 4,450 employees (gross sample) at the time of data collection. The written survey ended up with an average and quite satisfactory response rate of 41% and a sample of 1,840 hospital employees (net sample) who completed and returned the questionnaire focused on “work and health in hospitals”. No occupational group or hierarchical level was excluded from the survey. This means that the reason for nonparticipation was self-selection and not exclusion. However, the present study was restricted to 1,441 healthcare workers, excluding the commercial, technical, catering and other service staff.

## Measures

### Exposure or predictor variable(s)

#### Workplace violence and discrimination

Violence and discrimination at work was assessed by a 9-item measure taken from the Swiss Health Survey. The overall question “Have you experienced the following in the past 12 months at work?”, with a note that multiple answers were possible, was followed by nine different aspects and specific forms or types of workplace violence and discrimination, namely, five items on violence and aggression (verbal violence, threats and humiliation, physical violence, intimidation or bullying, sexual harassment) and four items on discrimination (disadvantage and unfair treatment due to age, gender, disability and nationality, ethnicity or skin colour). Three sum scales were constructed by simply counting or adding up the number of reported different kinds of violence and/or discrimination experienced at work (violence subscale, discrimination subscale, violence and/or discrimination full scale). The scales ranging from 0 to a possible maximum score of 4, 5 or 9 were then categorized into three groups, namely, “none” (0), “individual” (1) and “cumulated” (2 +) experienced forms or kinds of workplace violence and/or discrimination.

### Intervention variable

#### Work climate

A 6-item measure was used to assess the work climate, including five items on “social relations” and the “sense of community” at work taken from the Copenhagen Psychosocial Questionnaire (COPSOQ) [42]. The multi-item measure consists of the following six questions:

- Do you work isolated from your colleagues?
- Is it possible for you to talk to your colleagues while you are working?
- Is there a good atmosphere between you and your colleagues?
- Is there good cooperation between colleagues at work?
- Do you feel part of a community at your place of work?
- How often do you feel unfairly criticized or bullied by colleagues or superiors or exposed in front of others?

For each of the six items, five response options were given (always, often, sometimes, seldom, hardly ever or never), with scores ranging from 0 (always) to 4 (never) for the first and last item in the list and from 0 (never) to 4 (always) for the items between them. Then, a total score for the six reversely scored items was calculated and classified into four categories of the work climate, namely, poor (0–12), rather poor (13–15), moderate (16–18), and good (19–24).

### Outcome variables

#### Intention to quit one’s job

The turnover intention was measured by the following question and the corresponding answer (selected from three response options): “Have you ever seriously considered quitting and changing jobs since joining the company?” – “Yes, and since then, nothing has changed”.

#### Intention to leave one’s profession

The intention to leave one’s profession was measured by the following question and the corresponding response options: “How often have you been thinking about leaving the profession over the past 12 months?” – “Several times per month”, “Several times per week” or “Daily”. Those who reported “never” or only “several times per year” having thoughts about leaving the profession were categorized as not intending to leave.

### Control variables

#### Sex, age, education

The usual and most important sociodemographic characteristics were used as control variables and for statistical adjustment. Age in the survey was recorded not with an open-ended question and as a continuous variable (interval scale) for anonymity reasons but rather with a closed-ended question that asked the participants to choose one of five different age groups or categories (ordinal scale): < 25 years, 25–34 years, 35–44 years, 45–54 years, ≥ 55 years. Education was measured on a 12-point scale on which the participants were asked to note their highest educational attainment (ranging from “no compulsory education” to “university degree”); the responses were then categorized into four ordinally scaled levels of school and/or vocational education (low, medium, high, very high).

## Analysis

Stratified and profession-specific absolute and relative frequencies (number of cases, percentages) for all study variables were calculated to estimate the prevalence rates of experienced forms of workplace violence and/or discrimination, poor work climate and intentions to quit one’s job and leave one’s profession among different (groups of) health professions (Table 1).

**Table 1:** Relative and absolute frequencies of experienced forms of workplace violence and/or discrimination, the perceived work climate and intentions to change one’s job or leave one’s profession among health professionals (*N* = 1,441)

	**Nurses and midwives** (*n* = 882)	**Physicians** (*n* = 235)	**Therapists and other health professionals** (*n* = 324)	**All health professionals** (*n* = 1,441)
	% (n)	% (n)	% (n)	% (n)
**Forms of violence at work**				
• 0 (none)	79.6 (702)	82.6 (194)	88.3 (286)	82.0 (1,182)
• 1 (individual)	13.5 (119)	11.9 (28)	8.3 (27)	12.1 (174)
• 2–5 (cumulated)	6.9 (61)	5.5 (13)	3.4 (11)	5.9 (85)
**Forms of discrimination at work**				
• 0 (none)	91.2 (804)	86.4 (203)	88.0 (285)	89.7 (1,292)
• 1 (individual)	8.0 (71)	11.9 (28)	10.5 (34)	9.2 (133)
• 2–4 (cumulated)	0.8 (7)	1.7 (4)	1.5 (5)	1.1 (16)
**Forms of violence AND discrimination at work**				
• 0 (none)	75.1 (662)	75.3 (177)	79.0 (256)	76.0 (1,095)
• 1 (individual)	15.2 (134)	14.0 (33)	14.8 (48)	14.9 (215)
• 2–9 (cumulated)	9.8 (86)	10.6 (25)	6.2 (20)	9.1 (131)
**Work climate**				
• Poor (0–12)	3.4 (29)	6.5 (15)	4.5 (14)	4.1 (58)
• Rather poor (13–15)	15.4 (133)	18.2 (42)	26.0 (81)	18.2 (256)
• Moderate (16–18)	37.1 (320)	39.0 (90)	34.4 (107	36.8 (517)
• Good (19–24)	44.1 (381)	36.4 (84)	35.0 (109)	40.9 (574)
**Intention to change the job**				
• No / not anymore	83.4 (730)	87.4 (202)	81.9 (279)	83.7 (1,195)
• Yes	16.6 (145)	12.6 (29)	18.1 (43)	16.3 (232)
**Intention to leave the profession**				
• Never to occationally^a^	81.5 (712)	85.8 (199)	86.6 (279)	83.3 (1,190)
• Frequently^b^ to daily	18.5 (162)	14.2 (33)	13.4 (43)	16.7 (238)

^a^Several times a year

^b^Several times a month or week

**Fig. 2:**
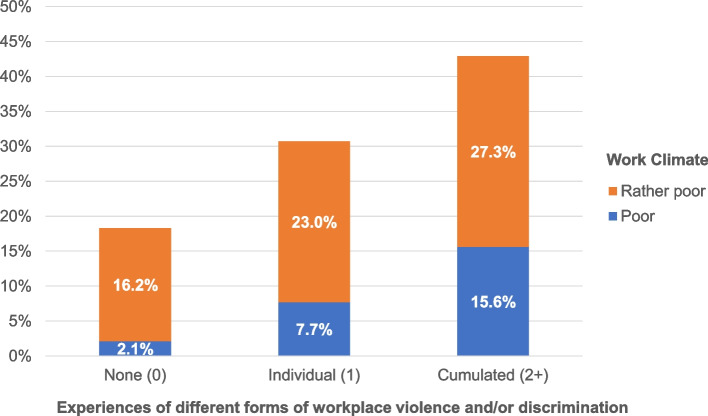
Proportions of health professionals who report a poor work climate, depending on the number of experienced forms of workplace violence and/or discrimination (*N* = 1,441)

**Fig. 3:**
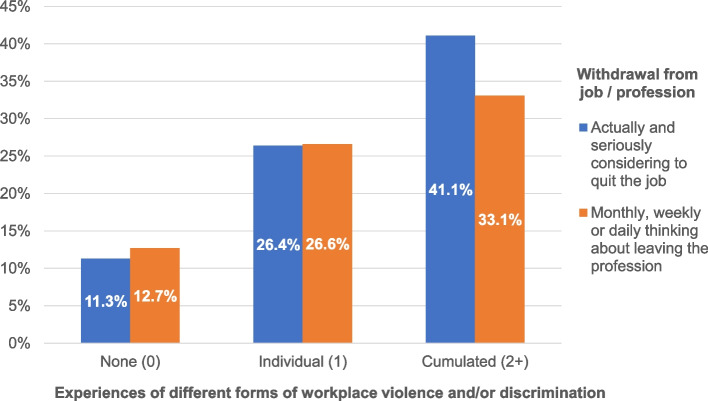
Proportions of health professionals who report intentions to quit their jobs and to leave their professions by numbers of experienced forms of workplace violence and/or discrimination (*N* = 1,441)

Then, prevalence rates or proportions of respondents with reported intentions to quit their job or leave their profession were calculated according to the cumulative numbers of experienced forms of workplace violence and/or discrimination categories and for the different groups of health professions separately (Tables 2 and 3). Additionally, bivariate associations or, more precisely, crosstabulations between the number of experienced forms of violence and/or discrimination and the proportion of respondents with poor work climate and with intentions to quit their job and leave their profession, were calculated and illustrated for the entire study population of health professionals (Figs. 2 and 3).

**Table 2:** Share and number of respondents who are *seriously considering to quit the job* by numbers of experienced forms of workplace violence and/or discrimination for different health professions and all health professionals (*N* = 1,441)

	**Nurses and midwives** (*n* = 882)	**Physicians** (*n* = 235)	**Therapists and other health professionals** (*n* = 324)	**All health professionals** (*n* = 1,441)
	% (n)	% (n)	% (n)	% (n)
**Study population**	**16.6** (145)	**12.6** (29)	**18.1** (58)	**16.3** (232)
**Experienced forms of violence at work**				
• None (0)	12.5 (87)	9.4 (18)	14.0 (40)	12.4 (145)
• Individual (1)	28.0 (33)	17.9 (5)	48.0 (12)	29.2 (50)
• Cumulated (2–5)	41.0 (25)	50.0 (6)	54.5 (6)	44.0 (37)
**Experienced forms of discrimination at work**				
• None (0)	14.7 (117)	10.6 (21)	15.2 (43)	14.1 (181)
• Individual (1)	35.7 (25)	25.0 (7)	42.4 (14)	35.1 (46)
• Cumulated (2–4)	42.9 (3)	25.0 (1)	20.0 (1)	31.3 (5)
**Experienced forms of violence AND discrimination at work**				
• None (0)	11.7 (77)	9.2 (16)	11.8 (30)	11.3 (123)
• Individual (1)	25.0 (33)	12.1 (4)	40.4 (19)	26.4 (56)
• Cumulated (2–4)	40.7 (35)	37.5 (9)	47.4 (9)	41.1 (53)

**Table 3:** Share and number of respondents who are *frequently or daily thinking about leaving the profession* by numbers of experienced forms of workplace violence and/or discrimination for different health professions and all health professionals (*N* = 1,441)

	**Nurses and midwives** (*n* = 882)	**Physicians** (*n* = 235)	**Therapists and other health professionals** (*n* = 324)	**All health professionals** (*n* = 1,441)
	% (n)	% (n)	% (n)	% (n)
**Study population**	**18.5** (162)	**14.2** (33)	**13.4** (43)	**16.7** (238)
**Experienced forms of violence at work**				
• None (0)	15.8 (110)	12.0 (23)	12.0 (34)	14.3 (167)
• Individual (1)	23.5 (28)	21.4 (6)	22.2 (6)	23.0 (40)
• Cumulated (2–5)	40.0 (24)	30.8 (4)	27.3 (3)	36.9 (31)
**Experienced forms of discrimination at work**				
• None (0)	16.4 (131)	11.5 (23)	10.6 (30)	14.4 (184)
• Individual (1)	42.9 (30)	28.6 (8)	32.4 (11)	37.1 (49)
• Cumulated (2–4)	14.3 (1)	50.0 (2)	40.0 (2)	31.3 (5)
**Experienced forms of violence AND discrimination at work**				
• None (0)	14.8 (97)	10.3 (18)	9.1 (23)	12.7 (138)
• Individual (1)	26.3 (35)	24.2 (8)	29.2 (14)	26.6 (57)
• Cumulated (2–9)	35.3 (30)	28.0 (7)	30.0 (6)	33.1 (43)

Finally, multivariate association analyses in the form of multiple logistic regressions were performed; in particular, four different increasingly specified and adjusted regression models were calculated to estimate independent, direct and indirect (mediated) predictive effects (odds ratios) of the cumulative number of experienced forms of workplace violence and/or discrimination on the outcome variables under study (Table 4).

**Table 4:** Associations between number of forms and combinations of workplace violence and discrimination, working climate and intentions to leave one’s job or profession among health professionals (*N* = 1,441)

	**Intention to quit the job** ^a^			**Intention to leave the profession** ^b^		
	% (n)	aOR^c^	95% CI	% (n)	aOR^c^	95% CI
**Total study population**	**16.3** (232)			**16.7** (238)		
MODEL 1						
**Experienced forms of violence at work**						
• None (0) (*n* = 1,182)	12.4 (145)	1		14.3 (167)	1	
• Individual (1) (*n* = 174)	29.2 (50)	2.86***	1.96–4.17	23.0 (40)	1.84**	1.24–2.74
• Cumulated (2–5) (*n* = 85)	44.0 (37)	5.74***	3.57–9.24	36.9 (31)	3.44***	2.12–5.60
No. of cases in model		1,396			1,397	
MODEL 2						
**Experienced forms of discrimination at work**						
• None (0) (*n* = 1,292)	14.1 (181)	1		14.4 (184)	1	
• Individual (1) (*n* = 133)	35.1 (46)	3.51***	2.36–5.24	37.1 (49)	3.39***	2.28–5.06
• Cumulated (2–4) (*n* = 16)	31.3 (5)	2.13	0.67–6.81	31.3 (5)	2.62	0.81–8.45
No. of cases in model		1,396			1,397	
MODEL 3						
**Experienced forms of violence AND discrimination at work**						
• None (0) (*n* = 1,095)	11.3 (123)	1		12.7 (138)	1	
• Individual (1) (*n* = 215)	26.4 (56)	2.87***	2.00–4.12	26.6 (57)	2.54***	1.78–3.63
• Cumulated (2–9) (*n* = 131)	41.1 (53)	5.39***	3.60–8.08	33.1 (43)	3.25***	2.14–4.93
No. of cases in model		1,396			1,397	
MODEL 4						
**Experienced forms of violence AND discrimination at work**						
• None (0) (*n* = 1,095)	11.3 (123)	1		12.7 (138)	1	
• Individual (1) (*n* = 215)	26.4 (56)	2.31***	1.58–3.37	26.6 (57)	2.07***	1.42–3.00
• Cumulated (2–9) (*n* = 131)	41.1 (53)	4.03***	2.62–6.21	33.1 (43)	2.52***	1.62–3.92
**Work climate**						
• Poor (0–12) (*n* = 58)	48.3 (28)	6.42***	3.38–12.2	37.9 (22)	4.15***	2.18–7.88
• Rather poor (13–15) (*n* = 256)	26.8 (68)	3.68***	2.40–5.64	24.3 (62)	2.82***	1.87–4.25
• Moderate (16–18) (*n* = 517)	16.1 (82)	2.16***	1.46–3.20	17.4 (89)	1.76**	1.22–2.53
• Good (19–24) (*n* = 574)	8.1 (46)	1		10.5 (60)	1	
No. of cases in model		1,363			1,363	

*p ≤ 0.05, **p < 0.01, ***p < 0.001

^a^Actually and seriously considering a job change

^b^Frequently thinking about leaving the profession during the past year (several times a month to daily)

^c^Odds ratios (OR) adjusted for control variables (sex, age, education)

## Results

Table 1 shows that discrimination at work was most commonly reported by physicians (14%), followed by therapists (12%) and nurses (9%), whereas different forms and self-reports of violence were most frequently reported among nurses (20%) compared to physicians (17%) or therapists and other health professionals (12%), who were less likely to report being victims of violence at work, as expected. This finding is largely in line with the international research literature, although in this study, population differences between the health professions or the three occupational groups are not strongly pronounced. In addition, differences between the two sexes across all occupations regarding such negative experiences at work cannot be observed or are hardly worth mentioning and are not found to be statistically significant.

In accordance with the high prevalence of experienced discrimination by colleagues, supervisors or the employer, a comparably high proportion of physicians (25%) reported working in a relatively poor climate (see Table 1). Only 19% of the nurses and midwives, but remarkably 31% of therapists and other health professionals, reported working in a poor climate. The intention to quit one’s job was accordingly most widespread among therapists (18%), followed by nurses and midwives (17%) and physicians (13%). Nurses, in contrast, clearly reported more frequently thinking about leaving their profession (19%) than did physicians (14%) or therapists and other health professionals (13%).

The more exposed health professionals are to different forms of violence and/or discrimination at the workplace, the more likely and frequently they were to seriously consider a job change (see Table 2). Likewise, repeatedly having thoughts about leaving one’s profession (several times a month to daily) was much more frequently reported among the health professionals who are more or most affected by one or more kinds and events of violence and/or discrimination at work compared to those who are not affected at all (see Table 3).

Despite different relative frequencies, the dose‒response relationship between such adverse events and bad experiences at work and the intentions to quit one’s job or leave one’s profession seems to be similarly pronounced between the different health professions and across the two studied intentions or outcomes. The multipliers or ratios of the proportions of potential job changers and career dropouts between the nonexposed and the most exposed and affected by different forms of violence and/or discrimination at work were found to lie almost consistently between a factor of 3 and 5 (see Tables 2 and 3).

As expected, experiences of workplace violence and/or discrimination and reports of a comparatively poor work climate or intentions to turnover and to leave go hand in hand or, more precisely, are closely related to each other. With an increasing number of different forms of violence and/or discrimination experienced at work, reports of poor work climate (see Fig. 2) and intentions to quit one’s job or leave one’s profession (see Fig. 3) among health professionals (and other hospital employees) increase as well and do so quite significantly. Only 18% of hospital employees in health professions who did not report having had any violent or discriminatory experiences at work in the past year compared to 43% of those who did report having had at least one such experience reported working in a poor climate (see Fig. 2). Only one out of eight or nine health professionals who did not experience any workplace violence or discrimination at all but more than one out of every three (approximately two-fifths) of those who are considered to be the most exposed to such issues (being affected by more than one form of violence and/or discrimination at work) reported seriously considering quitting their job or repeatedly thinking about leaving their profession (see Fig. 3). The relationships between exposure (reports and extents of workplace violence and/or discrimination) and outcomes (reports of poor work climate, intentions to change jobs or professions) were mostly found to be linear (clear gradient) and fairly strong (steep slope).

The results of multivariate association analyses or, more precisely, multiple logistic regression analyses (see Table 4) support the findings of the stratified bivariate association analyses (see Tables 2 and 3). There is a consistent clear and significant dose–effect relationship present between the number of kinds or forms of workplace violence and/or discrimination and the relative risk or likelihood of intending to quit one’s job or leave one’s profession among health professionals.

At first sight, experiences of violence at work seem to have a stronger impact or effect on such intentions or behaviours as quitting one’s job or leaving one’s profession than experiences of workplace discrimination (see Models 1 and 2 in Table 4). However, in consideration of the very low number of cases that reported having experienced more than one kind of discrimination at work, it was predictable and understandable that the odds ratio of a cumulated number of experienced forms of workplace discrimination had no significant effect on either considering a job change or thinking about leaving one’s profession (see Model 2 in Table 4). However, simply having experienced or been the victim of an individual form of discrimination was already associated with a strongly increased risk for such intentions or behaviours (OR > 3).

The association between the number of different violent and discriminatory experiences at work and the two outcome variables was found to be partly but not completely mediated by the work climate, which in turn is an additional, independent and strong predicting factor for the intention to quit one’s job or to leave one’s profession (see Models 3 and 4 in Table 4). Having experienced one or even more than one form of violence and/or discrimination at work was found to be associated with an up to fivefold increased risk of intending to change jobs or to leave one’s profession (see Model 3 in Table 4). However, independent of violent and/or discriminatory experiences at work, a poor work climate compared to a good one was found to be associated with a nearly threefold (and up to a more than sixfold) increased risk or chance for such thoughts of leaving one’s current position or career (see Model 4 in Table 4).

Overall, the relative risks of such negative workplace experiences (and a poor work climate) were found to be more accentuated for the intention to change one’s job than for the intention to leave one’s profession. This outcome could be reasonably expected since a job change is usually the more obvious, adequate feasible and more frequently chosen option or reaction than an occupational change or early retirement, as the latter reflect more serious and weighty decisions and consequences.

## Discussion

Although the serious and partly growing problems of workplace violence and discrimination in healthcare have received increased attention independent of one another in international, particularly in studies from North American and Asian countries [19, 24], the two phenomena are still largely unexplored in European and particularly German-speaking countries such as Switzerland, with few exceptions [25–27]. In Switzerland, as in many other countries, the numbers of cases of workplace violence and discrimination against health professionals are presumably strongly underreported; thus, the size of the problem is therefore either strongly underestimated or completely ignored. Correspondingly, the health- and particularly work-related consequences of such behaviours and incidents at work are little studied; they are especially poorly explored in health (services) research and among health professionals in Switzerland [27]. Even though in recent years aggressive and violent behaviours against or the unfair and discriminatory treatment of health professionals stemming from either patients and their relatives or from work colleagues and supervisors have been increasingly recognized and reported, they are still strongly underresearched; specifically, such incidents have not (yet) been closely examined with regard to work-related consequences such as turnover intentions or career endings.

The present study conducted among health professionals in selected public hospitals and rehabilitation clinics located in German-speaking Switzerland partly supports and partly supplements previous findings from international studies. Experiences of violence at work within the past year were found to be most prevalent among nurses and midwives (20%), whereas discrimination at work emerged as being most prevalent among physicians (14%). Intentions to change one’s job and leave one’s profession were more frequent among nurses (17%/19%) than among physicians (13%/14%). Therapists and other health professionals most frequently reported the intention to change their job (18%) and least frequently reported the intention to leave their profession (13%).

Some of these relative frequencies are not fully consistent with an earlier cross-sectional study conducted in 10 European countries among a large number of hospital nurses, which found for Switzerland that 28% of all participating nurses intended to leave their hospital workplace, while 6% reported considering leaving their profession [9]. However, our finding of one in eight or seven physicians intending to leave their job or profession is partly in line with a large Swiss cohort study that found that one in seven physicians (who graduated from a Swiss university medical school between 1980 and 2009) have left patient care [8].

Among all occupational groups (health professions), the prevalence of these intentions strongly increased with increasing numbers of experienced forms of workplace violence and/or discrimination, i.e., up to 38% or 28% (physicians), 41% or 35% (nurses) and 47% or 30% (therapists) in the cases of two or more than two experienced forms of violence and discrimination at work, respectively. A comparatively poor work climate was also found to be more likely with increasing numbers of having such negative experiences at work, reflecting increases of 18% (none) to over 31% (one) and 43% (more than one). A multivariate logistic regression analysis confirmed these results and revealed clear gradients and fairly strong adjusted effects of cumulative experiences of workplace violence and/or discrimination (aOR = 2.5–4.0) in the prediction of turnover intentions. However, the results also revealed a partial mediation by and a strong association with one’s work climate. These outcomes agree at least partly with a cross-sectional study conducted among foreign-born physicians in Finland that found a clear—although less strong-— positive or risk effect of discrimination (aOR = 1.6) and a significant negative or protective effect (aOR = 0.7) of a good team climate on turnover intentions [3].

A strong dose‒response relationship was observed between the number of experienced forms of workplace violence and/or discrimination and the chance or prevalence of the intention to change one’s job or leave one’s profession. In other words, the more exposed to or affected by workplace violence and/or discrimination a health professional is, the more likely and frequently he or she is to seriously consider a job change or repeatedly think about leaving his or her profession.

The strong and steady increase of such intended change of one’s job or profession with the increase in cumulated experiences of workplace violence and/or discrimination was consistently found in stratified bivariate association analyses and for all the main occupational groups of health professionals (nurses, physicians, therapists). Furthermore, this strong association and linear relation turned out to be similarly pronounced between these groups and was found for both types of exposure (being affected or threatened by violence at work, experiencing discrimination at work) and both outcomes under study (considering quitting one’s job, thinking about leaving one’s profession). This association was confirmed in multivariate regression analyses for the entire study population, even after adjustment for control variables and the potentially mediating variable of work climate. However, although the association was partly mediated by a poor work climate, the dose–effect relationships present between the numbers of forms of violence and/or discrimination experienced at work and the intention to quit one’s job or leave one’s profession, as expected, remained strong and highly significant.

In spite of such an evident, robust and strong relationship between experiences of workplace violence and/or discrimination and the intention to leave one’s job or profession, a poor work climate turned out to be another important and independent predictor or risk factor for such escape-focused reactions. However, workplace violence and discrimination was found to both directly and indirectly (via an impaired work climate) lead to escape plans, turnover intentions, job changes, career endings and early retirements.

### Limitations

The return or response rate of the postal survey among hospital employees was not too high but still in a normal range (in total about four in ten completed and returned the questionnaire). But the participation rate of the hospitals and clinics which were first contacted and asked to participate in the study and company survey was rather low (about one in ten hospitals agreed) but their selection was completely at random besides the mentioned inclusion criteria (language region, type of institution, size of workforce). The sample of the studied hospitals and clinics of course therefore is not representative for the entire healthcare sector in Switzerland. However, there is no indication of a selection bias due to systematic self-selection or self-exclusion (of hospitals and clinics) or non-responding (of individuals or employees).

Since all variables are observed and examined at the same time in cross-sectional studies with no need for follow-up, a temporal and causal link between exposure and outcome cannot be determined. Therefore, it is basically impossible to decide if there is a causal relationship between experiences of workplace violence and/or discrimination and intentions to quit one’s job or leave one’s profession.

In addition to this usual methodological limitation of cross-sectional data and related studies (no inferences or conclusions about any causal relationships can be drawn) and some other potential shortcomings of the study and the used data regarding the measures, the sampling and the selection of the survey or the study participants which have already been discussed in an previous publication (see [27]), it is difficult to say from a theoretical perspective if there is causality in some relationships, as illustrated in Fig. 1, or in which direction these relationships extend. The work climate in this study was considered to be a consequence rather than an antecedent of workplace violence and discrimination and an intervening variable that possibly and partly accounts for the relationship between experienced workplace violence and discrimination and the intention to change one’s job or profession. However, a good work climate might protect against or prevent unfair treatment or aggression from one’s colleagues (or supervisors). However, at the same time, it is not plausible that a good work climate hinders patients or their relatives from engaging in verbal abuse or other aggressive behaviours against healthcare workers.

## Conclusion

Every fourth health professional who participated in the study (or rather in the survey) has experienced and reported at least one form of violence and aggression (i.e., verbal and physical attacks from patients and/or relatives) and/or disadvantage and discrimination at work**.** At the same time, every sixth health professional has actually taken a job change into consideration and/or has regular thoughts of ending their career or changing professions. It seems that the common experiences of violence and/or discrimination at work—particularly when combined and cumulated but regardless of the form—are causing healthcare workers to have intentions (and engage in actions) to quit their job or leave their profession.

Being victimized and affected or threatened by violence at work stemming from patients or their relatives or being disadvantaged and discriminated against at work by one’s colleagues, supervisors, employers or corporate structures could be the main and true reasons or causes for the job changes or career endings frequently observed in healthcare settings or institution, especially among health professionals. These factors could be even more responsible for such behavior than the usual and well-studied suspected causes such as burnout [11, 12, 15, 17] and generally poor working conditions, rather high job demands and strong work stressors, such as work overload, long work hours, a low level of job control, work-privacy/family conflict or reward frustration and effort-reward imbalance [6, 11–14, 16, 43].

Job changes and career endings may be directly caused by work stress and burnout, as often reported and found. However, it can be assumed or seems reasonable to conclude that experiences of workplace violence and/or discrimination and the resulting poor work climate are “*the causes of the causes”* of job changes and career endings for health professionals. This relationship needs to be considered and addressed when trying to prevent or fight against high turnover rates, early retirements and staff shortages in the field of healthcare.

Finally, as the number of incidences of violence against healthcare workers increased strongly even before but particularly during and following the COVID-19 pandemic and crisis [44, 45], it can be reasonably expected that intentions (and decisions) to quit one’s job or leave one’s profession have occurred and will continuously occur even more frequently among healthcare workers and hospital employees, especially among health professionals who work in the field of direct patient care. This means that the problems of high turnover, career endings and understaffing in healthcare will likely become even more prominent in the future.

## Data Availability

Individual data were collected by random and full sample surveys among the workforces of several public hospitals and rehabilitation clinics. Data were collected anonymously and on a voluntary basis. However, data are not publicly accessible and freely available since the use and analysis of the pooled data and the publication of any research findings and study results out of it are restricted by contract with the participating companies (hospitals, clinics) to the University of Zurich (Epidemiology, Biostatistics and Prevention Institute) and the collaborating Careum Research, a division of the Careum Foundation. As contracted, the use of the data is basically limited to the two research institutions and disclosure and delivery of the data therefore is not permitted. In order to get an exceptional permission and possible conditional access to the survey data for scientific purposes the corresponding author as the principal investigator and the responsible for the data collection needs to be contacted.
